# Supplementary figures and images for: KLC4 shapes axon arbors during development and mediates adult behavior
Source: eLife. 2022 Oct 12;11:e74270. doi: 10.7554/eLife.74270 (PMC9596160; doi:10.7554/eLife.74270)

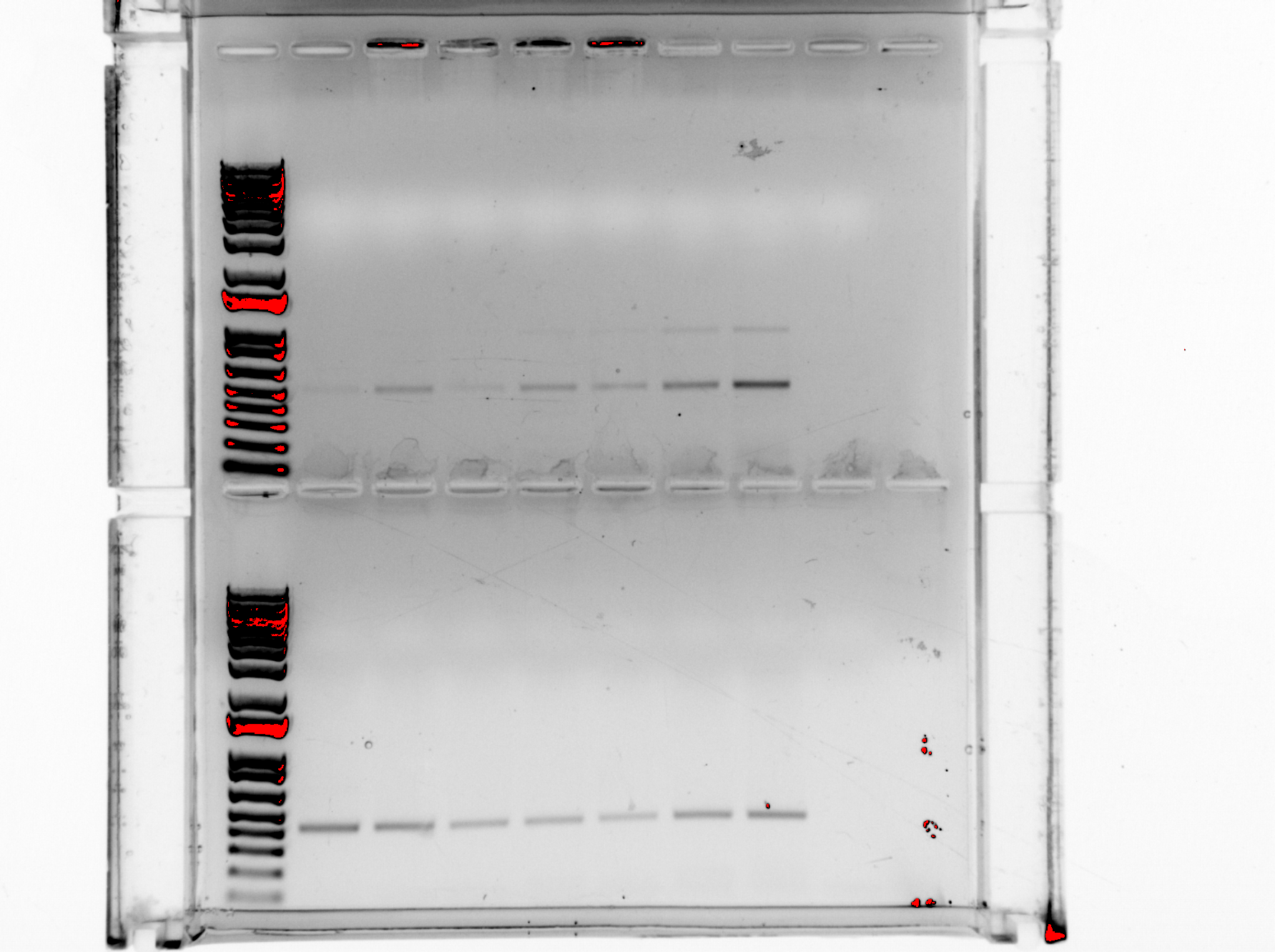

Supplement: Figure 1—source data 1. [file elife-74270-fig1-data1.zip › Figure 1 - Source Data 1.tif]
